# iPSC-derived familial Alzheimer’s *PSEN2*^*N141I*^ cholinergic neurons exhibit mutation-dependent molecular pathology corrected by insulin signaling

**DOI:** 10.1186/s13024-018-0265-5

**Published:** 2018-06-26

**Authors:** Cesar L. Moreno, Lucio Della Guardia, Valeria Shnyder, Maitane Ortiz-Virumbrales, Ilya Kruglikov, Bin Zhang, Eric E. Schadt, Rudolph E. Tanzi, Scott Noggle, Christoph Buettner, Sam Gandy

**Affiliations:** 1 Department of Neurology, NFL Neurological Care Center, Icahn School of Medicine at Mount Sinai, New York, NY 10029 USA; 2 Department of Psychiatry, Alzheimer’s Disease Research Center, Icahn School of Medicine at Mount Sinai, 5 E 98th St, New York, NY 10029 USA; 3 Department of Medicine, Diabetes, Obesity, and Metabolism Institute, Icahn School of Medicine at Mount Sinai, New York, NY 10029 USA; 4 New York Stem Cell Foundation Laboratories, New York, NY 10032 USA; 5 Icahn Institute and Department of Genetics and Genomic Sciences, Icahn School of Medicine at Mount Sinai, New York, NY 10029 USA; 6 Genetics and Aging Unit, Harvard Medical School, Massachusetts General Hospital, 114 16th Street, Charlestown, MA 02129 USA

## Abstract

**Background:**

Type 2 diabetes (T2D) is a recognized risk factor for the development of cognitive impairment (CI) and/or dementia, although the exact nature of the molecular pathology of T2D-associated CI remains obscure. One link between T2D and CI might involve decreased insulin signaling in brain and/or neurons in either animal or postmortem human brains as has been reported as a feature of Alzheimer’s disease (AD). Here we asked if neuronal insulin resistance is a cell autonomous phenomenon in a familial form of AD.

**Methods:**

We have applied a newly developed protocol for deriving human basal forebrain cholinergic neurons (BFCN) from skin fibroblasts via induced pluripotent stem cell (iPSC) technology. We generated wildtype and familial AD mutant *PSEN2*^*N141I*^ (presenilin 2) BFCNs and assessed if insulin signaling, insulin regulation of the major AD proteins Aβ and/or tau, and/or calcium fluxes is altered by the *PSEN2*^*N141I*^ mutation.

**Results:**

We report herein that wildtype, *PSEN2*^*N141I*^ and CRISPR/Cas9-corrected iPSC-derived BFCNs (and their precursors) show indistinguishable insulin signaling profiles as determined by the phosphorylation of canonical insulin signaling pathway molecules. Chronic insulin treatment of BFCNs of all genotypes led to a reduction in the Aβ42/40 ratio. Unexpectedly, we found a CRISPR/Cas9-correctable effect of *PSEN2*^*N141I*^ on calcium flux, which could be prevented by chronic exposure of BFCNs to insulin.

**Conclusions:**

Our studies indicate that the familial AD mutation *PSEN2*^*N141I*^ does not induce neuronal insulin resistance in a cell autonomous fashion. The ability of insulin to correct calcium fluxes and to lower Aβ42/40 ratio suggests that insulin acts to oppose an AD-pathophysiology. Hence, our results are consistent with a potential physiological role for insulin as a mediator of resilience by counteracting specific metabolic and molecular features of AD.

**Electronic supplementary material:**

The online version of this article (10.1186/s13024-018-0265-5) contains supplementary material, which is available to authorized users.

## Background

Type 2 diabetes (T2D) is associated with an increased risk for dementia in the setting of hyperinsulinemia and peripheral insulin resistance [15, 30]. There is as yet no consensus on whether the most important cause of dementia in the setting of T2D is related to Alzheimer’s disease (AD), vascular cognitive impairment and dementia (VCID) or some novel form of cognitive impairment with a predilection for a shared pathogenesis with T2D [13, 22, 26]. Furthermore, there is no consensus on the nomenclature for how these phenomena are described. The only exception is stroke, where it is accepted that the brain develops insulin resistance as a result of stroke [7] but even here, the brain regions, cell types, and intracellular bases of post-stroke insulin resistance are not well-established [25]. Other reports indicate that “brain insulin signaling” is diminished in patients with AD [3] and that insulin resistance and glucose hypo-metabolism seen in positron emission tomography (PET) brain scans predict cognitive outcome in humans [34]. There is substantial interest in the repurposing of intranasal insulin as a means of improving brain insulin signaling and thereby the cognitive function of both healthy individuals [4] and AD patients [6].

Given the poorly defined molecular pathology of brain insulin resistance in AD, we began by examining various potential mechanisms for the development of human brain insulin resistance as a cell autonomous phenomenon in neurons. Recently, an intriguing potential clue emerged in a report that γ-secretase hypofunction can underlie insulin resistance in adipocytes [27]. The most common mutations leading to familial Alzheimer’s disease (FAD) target the presenilin 1 (*PSEN1)* gene. Mutations in *PSEN1* or its homologue *PSEN*2 are associated with hypofunction of the γ-secretase leading to an increase in the amyloid β (Aβ) 42:40 ratio and Αβ42. The main aim of this study then was to test whether neuronal insulin signaling is impaired in neurons from subjects carrying FAD genes that encode hypofunctional γ-secretase and whether insulin resistance contributes to the phenotype of AD.

We analyzed the effect of insulin signaling on Aβ speciation as well as a battery of other determinations (such as electrophysiological profiling, toxin sensitivity, etc) in iPSC-derived wildtype and FAD mutant *PSEN2*^*N141I*^ neurons as well as their CRISPR/Cas9-corrected counterparts. We focused on a particular iPSC-derived neuronal subtype: the basal forebrain cholinergic neurons (BFCNs) [19] since these are believed to be one of the most vulnerable cells to early degeneration in AD. In many cases of early CI, the severity of the memory deficit appears to reflect the severity of the cholinergic deficiency [10, 11].

We observed that wildtype, *PSEN2*^*N141I*^ and CRISPR/Cas9-corrected iPSC-derived BFCNs (and their precursors) showed indistinguishable insulin signaling profiles as determined by the apparent phosphorylation stoichiometry of the canonical insulin signaling pathway molecules. Chronic insulin treatment of BFCNs of all genotypes led to a reduction in the Aβ42/40 ratio. Unexpectedly, a previously reported CRISPR/Cas9-correctable effect of *PSEN2*^*N141I*^ on calcium flux could be corrected by chronic exposure of BFCNs to physiologically stimulatory concentrations of insulin. Hence, the ability of insulin to correct calcium fluxes and to lower Aβ42/40 ratio suggests that insulin acts to oppose an AD-pathophysiology. Our results are consistent with a potential physiological role for insulin as a mediator of resilience by acting as an antagonist of specific metabolic and molecular features of AD.

## Results

### iPSC-derived neuroprecursors of BFCNs display dose-dependent insulin signaling

The cell lines used in this study are listed in Table 1. Three of these lines were derived from a cohort of siblings, two of whom carry the Volga German *PSEN2* ^N141I^ mutation [5] (AD1 and AD2) and a third who does not (fControl). Dedifferentiated fibroblasts were differentiated into iPSCs using non-incorporating modified mRNA transfer as described [21]. In addition, we also included a cell line that we have previously characterized from a healthy individual, which was reprogrammed using Sendai virus.

**Table 1:** Nomenclature and descriptions of iPSC lines used in this study

Line	Sex	*APOE*	Genotype	Reprogramming Method
Control	Male	E3/E3	Normal	Sendai virus
fControl	Female	E3/E3	Normal	mRNA Transfection
AD1	Female	E3/E4	*PSEN2* ^*N141I*^	mRNA Transfection
AD2	Female	E3/E3	*PSEN2* ^*N141I*^	mRNA Transfection
iAD1	Female	E3/E4	*Normal (Corrected AD1)*	mRNA Transfection
iAD2	Female	E3/E3	*Normal (Corrected AD2)*	mRNA Transfection

**Fig. 1:**
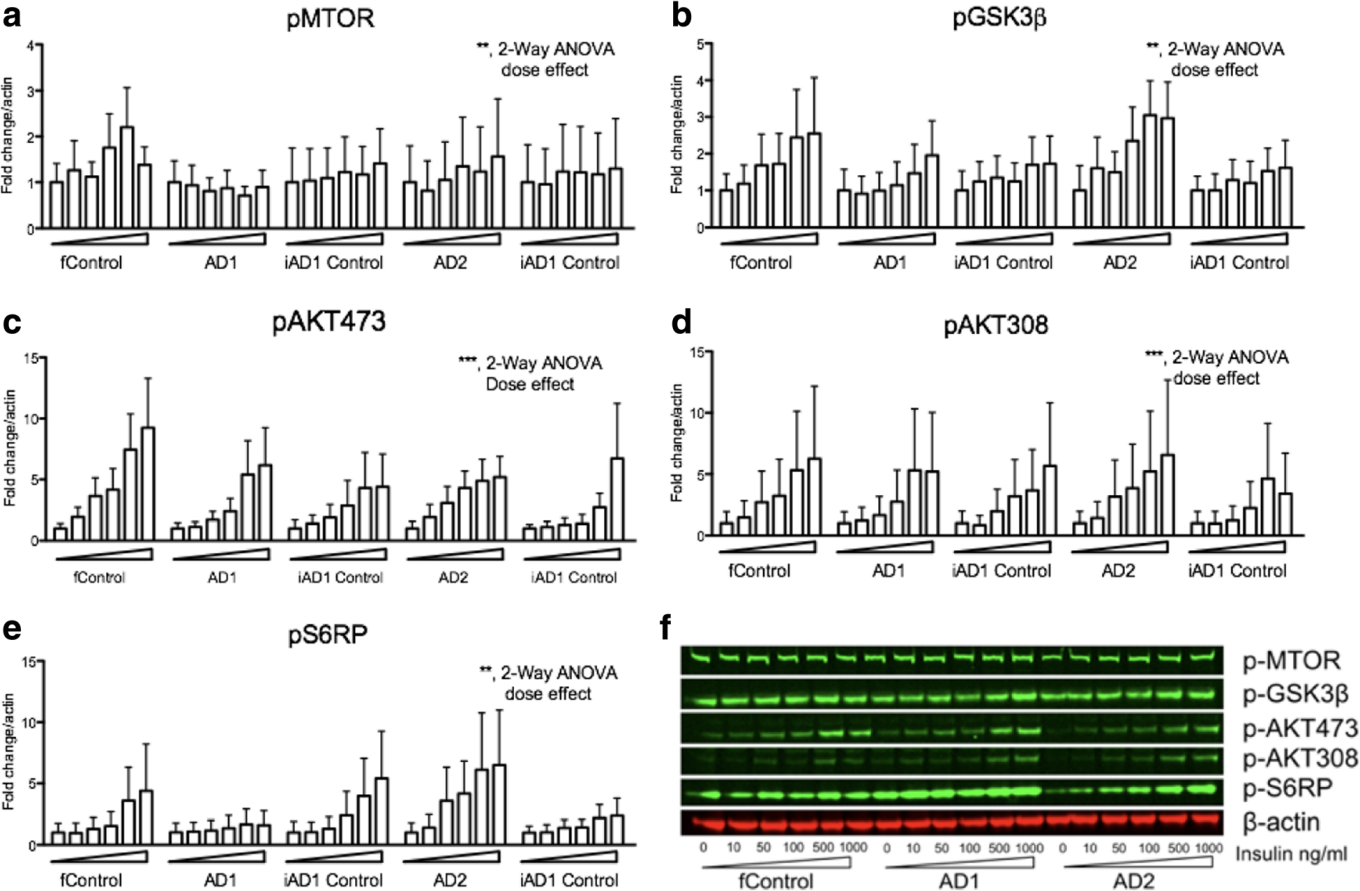
Downstream insulin signaling pathway molecules display similar phosphorylation status in iPSC-derived wildtype and *PSEN2*^*N141I*^ basal forebrain cholinergic neuroprecursors. **a**-**e** Western blot analysis of insulin signaling in iPSC-derived basal cholinergic neuroprecursor cell lines. Cells were insulin deprived overnight before the addition of corresponding insulin concentrations of 0, 10, 50, 100, 500, 1000 ng/ml. Lysates were collected after a 30-min exposure to the specified insulin concentration. Quantified western blot data was normalized to an actin standard and expressed as fold change to the 0 ng/ml vehicle control. Dose dependency was detected by 2-way ANOVA where indicated. These data correspond to results of three independent experiments. ** *P* < 0.01; *** *P* < .001. **f** Representative blots from a single experiment showing three of the lines

To characterize the insulin signaling of iPSC-derived wildtype and *PSEN2*^*N141I*^ neurons, we first conducted pilot studies to identify the optimal response time to achieve a maximal insulin signaling response. We found that after an overnight incubation without insulin, cells were responsive to a dose of 1000 ng/ml (~ 172 nM) after 10 and 30 min as assessed by AKT activation (see Additional file 1: Figure S1). We therefore used the 30-min incubation time to study phosphorylation of downstream molecules in the insulin signaling pathway. We next determined an insulin dose- response in neuroprecursor cells for BFCNs (Fig. 1).

Western blot analyses indicated that insulin activated all the canonical targets at the concentration employed. For reference, the dose range utilized ranged from reported physiological levels [32] (10 ng/ml is approximately 1.72 nM) to hyperphysiological levels (1000 ng/ml). Note that our highest insulin dose, 172 nM, is comparable to Kahn’s standard dose of 100 nM that was used to study insulin signaling in iPSC-derived myotubes [12]. We found that the effect of insulin was most robust on the phosphorylation of AKT (Fig. 1c and d), which is a key signaling node within the PI-3 K signaling pathway. Downstream of AKT, glycogen synthase kinase-3 β (GSK3β) and ribosomal protein S6 (pS6RP), which mediates the translational regulation through insulin were activated by insulin. Similarly, insulin phosphorylated mammalian target of rapamycin complex (pMTOR) in a dose responsive fashion [31]. Importantly, insulin signaling was not reduced in neuroprecursor lines that carried the FAD-mutation. Thus, the insulin resistance effect of hypofunctional γ-secretase as reported in adipocytes [27] was not readily detectable in our iPSC-derived BFCNs harboring hypofunctional γ-secretase due to familial AD mutant *PSEN2*^*N141*^.

### iPSC-derived BFCNs display dose-dependent insulin signaling

**Fig. 2:**
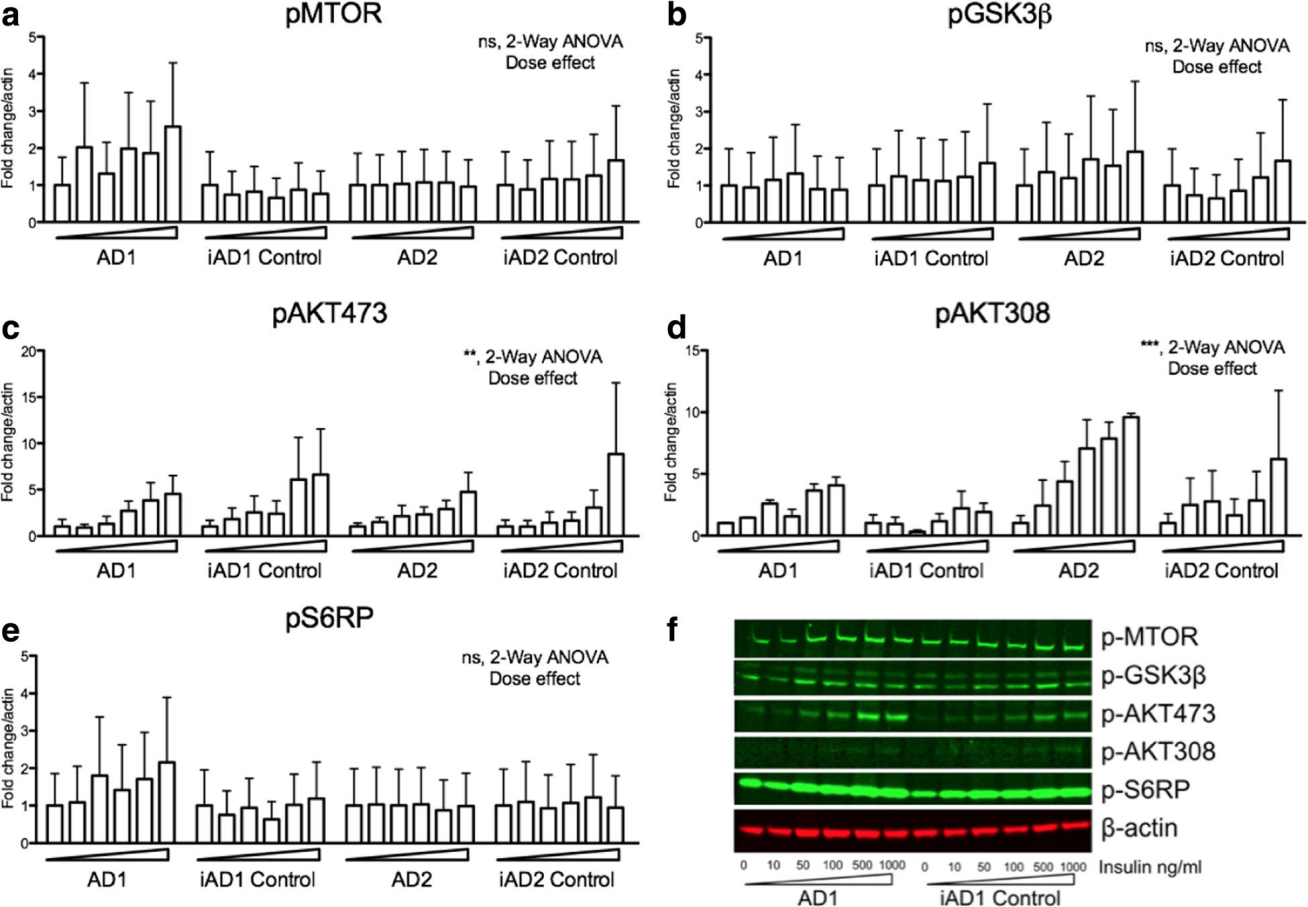
Downstream insulin signaling pathway molecules display similar status of phosphorylation in iPSC-derived wildtype and *PSEN2*^*N141I*^ basal forebrain cholinergic neurons (BFCNs). **a**-**e** Western blot analysis of iPSC-derived BFCNs. Cells (DIV 34) were insulin deprived overnight before the addition of insulin at 0, 10, 50, 100, 500, 1000 ng/ml. Cells were collected after a 30-min exposure. Quantifications of western blot data is normalized over actin and expressed as fold change to vehicle treated cells. These data correspond to results of two to three independent experiments. Dose dependency was detected by 2-way ANOVA where indicated. ** *P* < 0.01; *** *P* < .001. **f** Representative blots from a single experiment showing three of the lines

Different transcriptomic signatures and protein markers are observed through the neuronal differentiation process in iPSCs, corresponding with neurodevelopmental observations seen in vivo [28]. We have detected similar processes in the differentiation of our BFCNs [19]. Since insulin/insulin-like growth factor signaling exerts pleiotropic effects during neurodevelopment, we next studied whether insulin signaling differed as a function of the different developmental stages of the BFCN lineage. For this and all subsequent experiments, we compared the FAD-related lines with their engineered isogenic controls, due to the inherent variations we had observed across cell lines in our studies with the neuroprecursor cell lines (Fig. 1). We found similar molecular responses to insulin, whereby the proximal insulin cascade signatures i.e. pAKT were most robustly induced (Fig. 2). These findings demonstrated the integrity of insulin signaling via AKT and downstream signaling pathways such as s6RP in iPSC-derived BFCNs. However, we were unable to identify FAD-related differences in insulin signaling in the iPSC-derived BFCNs from *PSEN* mutation carriers.

### Insulin regulates Aβ production by BFCNs

**Fig. 3:**
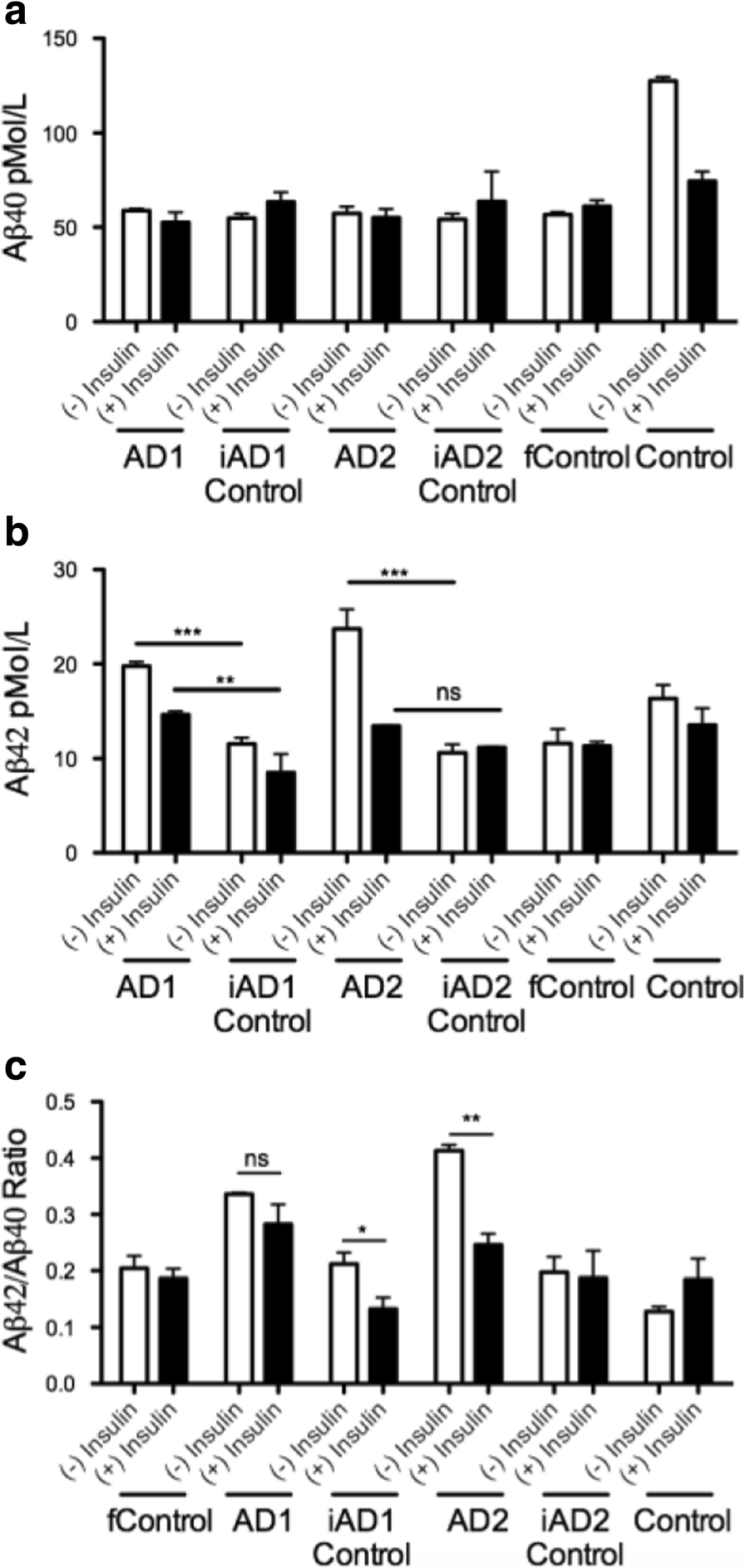
Chronic insulin administration reduces the Aβ42/40 ratio in the conditioned media of BFCNs harboring FAD mutations. ELISA quantification of iPSC derived basal cholinergic neurons measuring amyloid β. Cells (DIV 34) were insulin deprived for 3 days or given 1000 ng/mL every 24 h for 3 days. Aβ 40 (**a**), Aβ 42 (**b**), and Aβ 42/40 (**c**) measured in collected media. These data correspond to results of 2 independent experiments. * *P* < 0.05; ** *P* < .01

In order to determine whether insulin signaling could affect the metabolism of any of the hallmark proteins involved in AD pathology, we investigated if Aβ production was affected by chronic insulin exposure. In the study by Ortiz-Virumbrales and colleagues [19], we showed that by correcting the Volga German point mutation in *PSEN2*, the Aβ 42/40 ratio was normalized. We next tested whether insulin could modulate the Aβ 42/40 ratio in the cell lines that we were studying. Indeed, the Aβ 42/40 ratio that accumulated in the media being normalized in the corrected clones (e.g. AD1 vs iAD1 Control) (Fig. 3a-c). This effect was modulated by insulin in a cell-line-specific manner; for example, a significant reduction of Aβ 42/40 ratio by insulin was seen in the iAD1 control and the AD2 line. Hence, insulin tended to reduce the Aβ 42/40 ratio regardless of whether the ratio was elevated or not suggesting that insulin exerts anti-amyloidogenic effects, which in turn would be consistent with a role for insulin in preventing cerebral amyloidosis.

Using the same experimental design, we tested the status of tau phosphorylation at Ser^202^/Thr205 (Additional file 1: Figures S2a, 2b), but failed to detect FAD mutation-related changes in tau phosphorylation.

### Insulin promotes Ca^2+^ flux in iPSC-derived BFCNs

**Fig. 4:**
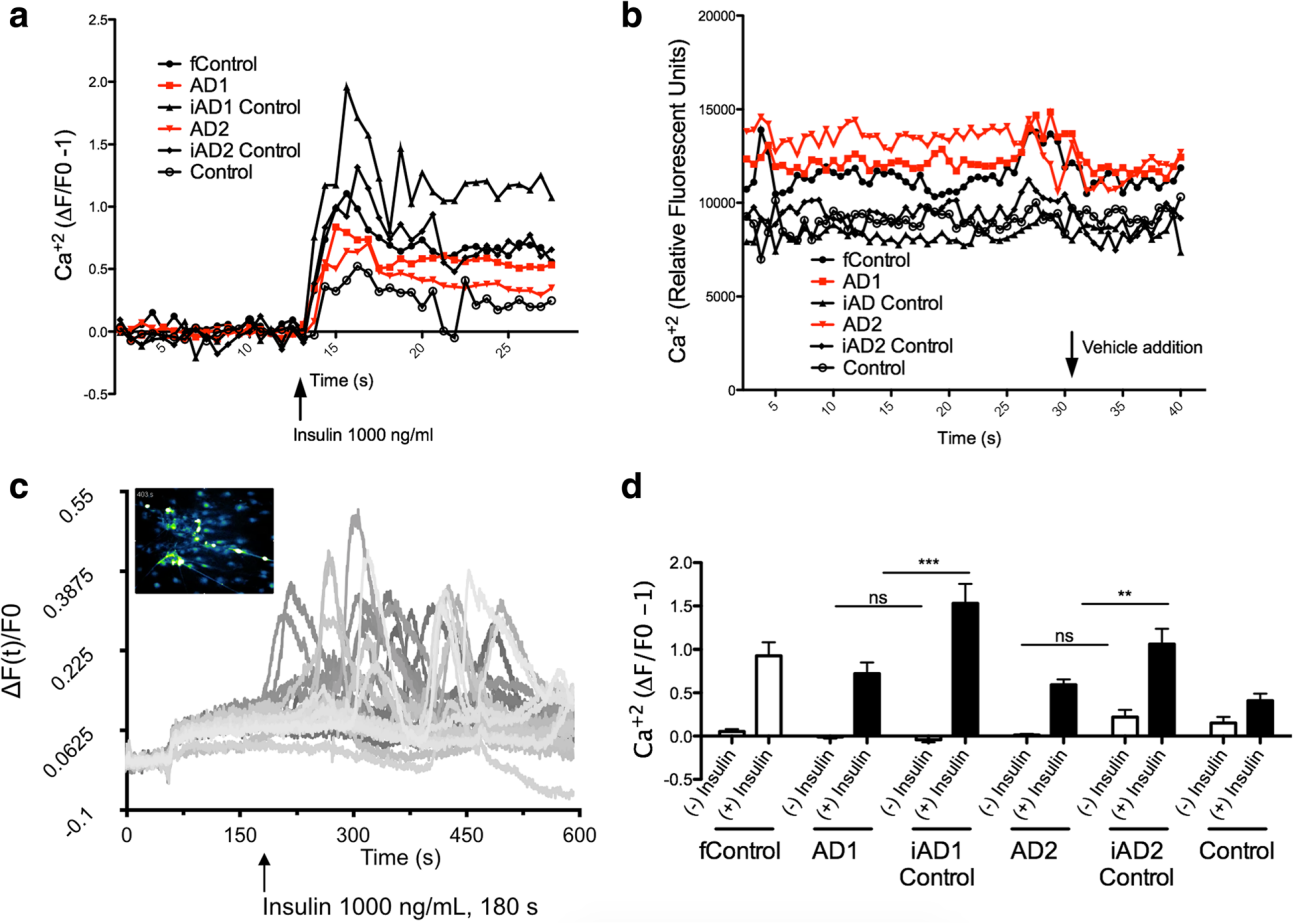
Insulin promotes Ca^2+^ flux in iPSC-derived BFCNs. Cells cultured in 96-well plates were insulin deprived overnight before insulin or vehicle was added to a final concentration of 1000 ng/ml after 25 s of baseline recording (data from a total of 4 experiments). **a** Calcium response to insulin normalized to 20 s of baseline recording before insulin addition. **b** Calcium response to addition of vehicle recorded as arbitrary relative fluorescent units. **c** Visual corroboration and quantification of Ca^2+^ in individual cells. **d** Average normalized calcium responses for baseline, (−) Insulin, and response, (+) insulin, in IPSC-derived basal cholinergic neurons. An effect by insulin exposure was detected by 2-way ANOVA (*p* < .0001). Bonferroni comparisons ***, *P* < .001; ** *P* < 0.01

As reported by Ortiz-Virumbrales and colleagues [19], iPSC-derived BFCNs from FAD mutation carriers showed reduced excitability and decreased spike amplitudes. We tested whether insulin could stimulate Ca^2+^ activity, as a proxy for electrophysiological activity, in iPSC-derived neurons and whether any changes that we detected would be affected by the presence of the mutant FAD gene. We recorded Ca^2+^ flux as measured by relative fluorescent units (Fig. 4) and observed that insulin robustly increased Ca^2+^ mediated fluorescence in all cell lines tested (Fig. 4a), compared to vehicle treatment (Fig. 4b). We obtained additional visual corroboration of Ca^2+^ activity (Fig. 4c and Additional file 2: Video 1). In conclusion, we observed that all cell lines tested displayed enhanced calcium-mediated fluorescence following insulin treatment.

## Discussion

Alzheimer’s disease (AD) pathology is characterized by synaptic and neuronal degeneration together with accumulation of extracellular plaques composed of Aβ [17], as well as neurofibrillary tangles composed of hyper-phosphorylated tau [16]. In terms of molecular pathogenesis, Aβ accumulation can be directly linked to some mutations leading to early onset FAD. However, in the vast majority of cases, i.e. in sporadic AD, the disease may begin with inflammation or lipid abnormalities and the plaques and tangles arise only later as the disease progresses [14]. Multiple co-existent pathologies (vascular, synuclein, etc.) can further complicate the picture.

The causality dilemma is illustrated by evidence linking AD to insulin resistance. An early analysis of this relationship comes from the Rotterdam study where a significant association of T2D and dementia believed to be AD was reported [20]. Additional epidemiological studies also reported similar links between T2D and dementia believed to be AD [1, 35], while others have observed that cognitively normal patients with T2D can show reduced cerebral glucose metabolism [2]. The latter observation could potentially dovetail with the fact that most dementia phenotypes are preceded by cerebral glucose hypo-metabolism [34], but it is not at all clear that all T2D patients develop cognitive decline. These studies support the notion that AD and T2D may have additive or synergistic effects on brain glucose metabolism. This hypothesis however is limited in its ability to elucidate the exact nature and molecular underpinnings of the relevant neuropathology.

At least three studies using post-mortem brains have suggested that AD (or cognitive impairment) is associated with impaired insulin signaling. In one study, AD brains were reported to display changes in the distribution of the insulin receptor as well as a reduction in IRS-1 and IRS-2, supporting the notion that insulin signaling would be impaired in those brain regions [18]. These observations complemented previous reports showing comparable reductions in mRNA levels within the insulin/IGF pathways [29]. However, none of these data substitute for a functional assessment of insulin signaling. The more definitive test of impaired insulin signaling was performed by Talbot et al.*.* using postmortem brain slices from subjects that were cognitively impaired or with AD, demonstrating that insulin signaling was indeed impaired in neuronal populations [32]. Taken together, these observations support the hypothesis that AD (and cognitive impairment) may be associated with disrupted insulin signaling in the brain.

Alternatively, some studies also suggest that AD is the causative or exacerbating factor for T2D [13, 35]. In this context, it is important to point out that brain insulin signaling controls systemic metabolism by regulating autonomic outflow from the brain to visceral organs such as liver and adipose tissue. The experimental induction of insulin resistance in a variety of brain regions and neuronal cell types has been found to impair metabolic control and increase the susceptibility to metabolic disease [23]. Hence, a disruption of insulin signaling along with AD pathology in brain regions controlling metabolism, such as the hypothalamus, can increase the susceptibility to diabetes and underpin the association of AD with T2D. For example, our group recently showed that *APP/PSEN1* amyloid-depositing mice exhibit reduced hypothalamic insulin signaling and are more susceptible to aging and over-nutrition induced prediabetes [24]. It is possible that hypothalamic amyloidosis may contribute to this phenomenon and may further potentiate T2D due to reduced brain insulin signaling [32]. A study by Calon et al summarizes this reciprocity by showing an enhanced sensitivity of triple transgenic amyloid-depositing mice to high fat diet and a reversal of memory deficits and decrease in soluble Aβ following insulin administration [33]. These observations suggest that AD and T2D pathologies may be able to set up a self-perpetuating “vicious cycle” with one another.

At this point, the key questions are: how exactly does AD impair brain and neuronal insulin signaling and if neuronal insulin resistance is induced by AD pathology, is it a cell autonomous effect or secondary to altered in-vivo – including paracrine – regulation. As an example of paracrine regulation of insulin signaling, a recent study demonstrated that ApoE4 secreted from astrocytes binds to the insulin receptor and may reduce insulin signaling by trapping the insulin receptor in the endosome [36]. FAD could similarly impair insulin signaling through altered paracrine signaling or indirect effects in the microenvironment resulting in available cytokines or metabolites. The fact that we did not find insulin signaling to be altered in neurons derived from several FAD forms supports this notion. Future studies that examine such paracrine signaling in co-culture models of astrocytes or glia cells with neurons will examine these possibilities.

In an earlier study, we generated basal cholinergic neurons from iPSC lines carrying the *PSEN2*^*N141I*^ mutation, along with corresponding familial and isogenic controls [19]. In the current study, we observed that both cholinergic neuroprecursors and BFCNs derived from iPSCs exhibit classic responses to insulin that are not affected by the presence of AD mutations.

We went on to test whether chronic exposure to insulin could affect the production of Aβ. Previous pulse-chase studies demonstrated that insulin reduces intracellular amyloid beta production by modulating βAPP trafficking [9]. We observed a bimodal response where chronic insulin exposure resulted in the ostensibly beneficial reduction of the Aβ42/40 ratio. These results support the existence of additional mechanisms that synergize with insulin action in its potential regulation of amyloid production. These data correlate well with results from a human clinical trial using intranasal insulin administration in patients with mild cognitive impairment and mild to moderate AD. Unbiased exploratory analyses revealed that some subtle memory benefits resulted and that the memory changes were associated with changes in Aβ42 [6]. These observations merit additional studies aimed at identifying factors that interact with insulin in modulating brain Aβ42, and to study the underpinnings generating the variabilities in Aβ42/40 that have been observed in our lab and in other labs using iPSC models of AD [8].

Finally, in our earlier study [19], we reported that electrophysiological parameters such as action potential frequency and amplitude were reduced in cells carrying the *PSEN2* ^*N141I*^ mutation. Therefore, we decided to assess whether insulin could modulate calcium transients as a reflection of electrophysiological effects. We found that insulin promotes Ca^2+^ mediated-fluorescence in all lines tested, and that *PSEN2* mutant lines show reduced responses compared to their isogenic control lines. These changes were normalized either when the mutation was corrected with CRISPR/Cas9 gene editing or when insulin was chronically administered, apparently leading to a physiological correction by promoting Ca^2+^ currents that enhanced synaptic transmission [37]. These observations warrant future patch-clamp studies to best illustrate the nature of Ca^2+^ deficiencies in response to insulin. Furthermore, future studies would benefit by the inclusion of corresponding isogenic controls i.e., with engineered FAD mutations. These studies would help explain if the deficiency Ca^2+^ flux in the insulin-mediated response of the unrelated control line (which was lower than in all other lines), is a result of genetic background, and if FAD mutations would further dampen such responses.

## Conclusions

In summary, wildtype, *PSEN2*^*N141I*^ and CRISPR/Cas9-corrected iPSC-derived BFCNs (and their precursors) showed indistinguishable insulin signaling profiles as determined by the apparent phosphorylation stoichiometry of the canonical insulin signaling pathway molecules. Chronic insulin treatment of BFCNs of all genotypes led to a reduction in the Aβ42/40 ratio. Unexpectedly, a previously reported CRISPR/Cas9-correctable effect of *PSEN2*^*N141I*^ on calcium flux could be corrected by chronic exposure of BFCNs to physiologically stimulatory concentrations of insulin. Taking this calcium flux-correction effect of insulin together with its Aβ42/40 ratio-lowering effect suggests that insulin action at each of these steps acts to oppose an AD-related phenomenon. Our results are consistent with a potential physiological role for insulin as a mediator of resilience by acting as an antagonist of specific metabolic and molecular features of AD.

## Methods

### Cell derivation and maintenance

All iPSC lines utilized in these studies were originally obtained from the NYSCF Repository and were propagated according to standard guidelines [21]. The lines used were designated 28,945 (Control), 948 (AD1), 949 (fControl), and 950 (AD2). Additional clones with single point mutation corrections were generated from AD1 and AD2 using CRISPR technology as described [19]. Briefly, the CRISPR/Cas9 system was engineered to target the *PSEN2*^N141I^ locus by cloning a single guide RNA (sgRNA) into pSpCas9(BB)-2A-GFP (PX458). Subsequently, the cells were transfected with the vector and a complementary ssODN by electroporation to serve as a correctional template via homology-directed repair (HDR). All iPSC cell lines were expanded and maintained in serum-free mTeSR1 media (Stem Cell Technologies) in a feeder-free system using Cultrex (Trevigen). Media was supplemented with 10 μM ROCK inhibitor (Y27632, Stemgent) during cell passages.

### BFCN differentiation

The differentiation protocol has been described in detail in [19]. Briefly, iPSC lines grown in mTeSR1 media were dissociated using Accutase (Sigma-Aldrich) and plated onto Cultrex (Trevigen) coated 6-well plates at a density of 400,000–1,000,000 cells per well. The cells were allowed to reach full confluency in mTeSR1 media before initiating the differentiation process. At the beginning of differentiation (day 0) and continuing to day 8, cells were maintained in Custom mTeSR1 media (Stem Cell Technologies) lacking pluripotency promoters (i.e., bFGF, TGF-Beta, Li-Cl, GABA and pipecolic acid). Starting on day 2, the media was supplemented with dual SMAD inhibitors (SB431542 10 μM plus LDN193189 250 nM, Selleck Chem) as well as the ventralizing agents SAG at 500 nM (R&D) and Purmorphamine at 2 μM (Stemgent). During this period of induction, the media were changed every other day. On day 9, the media were gradually switched to Brainphys media (Stemcell Technologies) supplemented with B27 (Life Technologies). At the end of this period, neural progenitors were generated and used in subsequent assays. Alternatively, neuroprogenitors were dissociated using Accutase and seeded in plates coated with branched polyethynilimine (0.1%, Sigma-Aldrich) and laminin (10 mg/mL, Life Technology) in Brainphys media + B27 supplement with nerve growth factor (NGF) (Alamone labs, 50 ng/mL) and brain derived neurotrophic factor (BDNF) (R&D, 50 ng/mL). For this protocol, the media were changed every 2 days until analysis.

### Insulin administration

Cells were cultured overnight using media supplemented with B27 without insulin (Thermofisher A1895601). After the overnight culture, insulin (Sigma Cat. 91077C) or vehicle was added to the media as indicated. Insulin was prepared fresh from frozen stocks every day before administration. For chronic studies, a daily dose of 1000 ng/ml of insulin or vehicle (PBS) was added.

### Protein extraction and western blot analysis

Cells were washed with cold PBS and then lysed with cold RIPA buffer (Thermo Fisher Scientific) supplemented with a cocktail of phosphatase and protease inhibitors (Life Technologies). The cells were scraped from the plates and passed through a 23-gauge needle four times before centrifuging at 12000RPM for 20 min at 4 °C. After centrifugation, the supernatant was collected and protein concentration was measured using a BCA or a Bradford quantification assay (Thermo Fisher Scientific). Equal amounts of protein lysates were loaded into NuPage BisTris 4–12% gradient gels (Thermo Fisher Scientific) and then transferred to 0.45 μm PVDF membranes (Millipore). The membranes were blocked with TBS and Odyssey blocking buffer (LI-COR) for one hour at room temperature followed by an overnight incubation with primary antibodies at 4 °C. The following antibodies were used: phospho-mTOR (#2971), phospho-AKT (Thr308) (#4056), phospho-AKT (Ser473) (#9271), phospho-GSK-3β (Ser9) (#9323), phospho-S6-Ribosomal Protein (Ser235/236) (#2211) from Cell Signaling Technology; phospho-Tau (Ser202/Thr205) (MN1020) from Thermo Fisher Scientific; and β-Actin (sc-81,178) from Santa Cruz Biotechnology or Gapdh (g8795) from Sigma-Aldrich. After overnight incubation, membranes were washed 3X with TBS-Tween and stained for one hour with secondary antibodies- IR-Dye 800 Goat Anti-rabbit (LI-COR) and Dylight 680 Goat Anti-mouse (Thermo Fisher Scientific) – diluted at room temperature in 0.01% SDS TBS-Tween. Finally, the membranes were washed 4 times and scanned using Odyssey Classic (LI-COR). Image Studio Software (LI-COR) was used to quantify bands.

### Calcium image staining and microfluorimetry in response to insulin

Cells were deprived of insulin for a period of 24 h as previously described, and then incubated with a 2.5 ng/μl solution of Fluo-4, AM in 0.01% Pluronic F-127 (Life Technologies) dissolved in cell media (1:1000) for 15 min at 37 °C. The cells were then washed twice with media and cultured with a Brainphys media lacking Phenol Red (Stem Cell Technologies) for an additional 15 min before recording fluorescence. For microfluorimetry, relative fluorescent units (RFUs) were obtained using a Flexstation 2 (Molecular Devices Corp.) that was set to record RFUs in a 96-well format for a period of 70 s in 0.6 s intervals using Softmax Pro software (Molecular Devices Corp.). The wavelengths used were - excitation at 485 nm, emission at 538 nm and cut-off at 530 nm. The RFUs were exported using Softmax Pro (Molecular Devices) and after recording the baseline fluorescence, the cells were treated with 1000 ng/ml insulin or vehicle for 25 s. Visual corroboration of Ca^2+^ activity in individual cells was obtained using coverslips that were transferred to a recording chamber mounted on an upright Olympus BX61 microscope and fluorescence was recorded at 2 Hz by a cooled CCD camera (Hamamatsu Orca R2). A baseline recording of transients was obtained for the first 180 s before insulin application (1000 ng/ml) via whole chamber perfusion at room temperature for a period of 420 s. Ca^2+^ transients were expressed in a form of ΔF(t)/F0, where F0 is a baseline fluorescence of a given region of interest and ΔF is a difference between current level of fluorescence F(t) and F0. Fluctuations of ΔF(t)/F0 that were less than 0.05 were considered as non-responses.

### Enzyme-linked immunosorbent assay (ELISA)

Cells were plated in a 24-well plate with Brainphys media supplemented with B27 (minus insulin) and treated with 1000 ng/ml of insulin or equivalent volume of PBS daily for 3 days. On the third day, the culture media were collected 30 min after insulin administration and ELISAs were performed on these aspirates using β-Amyloid 40 and 42 kits (WAKO Chemicals) according to manufacturer’s instructions.

### Statistical analysis

Western blot experiments, Aβ42/40 ELISAs and relative fluorescent units (RFUs) or ΔF(t)/F0 were analyzed for statistical significance using 2-Way ANOVA followed by Bonferroni post hoc tests. All statistical analysis was computed using PRISM 5. *, *p* < .05; **, *p* < .01; ***, *p* < .001.

## Additional files

Additional file 1: **Figure S1.** Western blots of iPSC-derived basal cholinergic neuroprecursors cell lines. Cells were insulin deprived overnight before the addition 1000 ng/ml of insulin (insulin was not added to time 0′). Lysates were collected at 0, 10, or 30-min exposure. Quantified western blot data was normalized over Gapdh and expressed as fold change of 0 ng/ml dose. An effect on concentration response was detected by 2-way ANOVA if indicated. These data correspond to results of three independent experiments. ** *P* < 0.01; *** *P* < .001. (H) Representative blots from a single experiment showing three of the lines. **Figure S2.** (A) Western blot quantification of iPSC derived basal cholinergic neurons (two independent experiments). Cells (DIV 34) were insulin deprived for 3 days or given 1000 ng/mL every 24 h for 3 days. (B) Representative blots from a single experiment. (DOCX 262 kb)

Additional file 2: Video 1. Supplemental Video. (M4V 7249 kb)
