# Supplementary material for: iPSC-derived familial Alzheimer’s PSEN2N141I cholinergic neurons exhibit mutation-dependent molecular pathology corrected by insulin signaling
Source: Mol Neurodegener. 2018 Jun 26;13:33. doi: 10.1186/s13024-018-0265-5 (PMC6020427; doi:10.1186/s13024-018-0265-5)
Supplement: Supplementary file 1 — Figure S1. Western blots of iPSC-derived basal cholinergic neuroprecursors cell lines. Cells were insulin deprived overnight before the addition 1000 ng/ml of insulin (insulin was not added to time 0′). Lysates were collected at 0, 10, or 30-min exposure. Quantified western blot data was normalized over Gapdh and expressed as fold change of 0 ng/ml dose. An effect on concentration response was detected by 2-way ANOVA if indicated. These data correspond to results of three independent experiments. ** P < 0.01; *** P < .001. (H) Representative blots from a single experiment showing three of the lines. Figure S2. (A) Western blot quantification of iPSC derived basal cholinergic neurons (two independent experiments). Cells (DIV 34) were insulin deprived for 3 days or given 1000 ng/mL every 24 h for 3 days. (B) Representative blots from a single experiment. (DOCX 262 kb) [file 13024_2018_265_MOESM1_ESM.docx]

**Additional file**


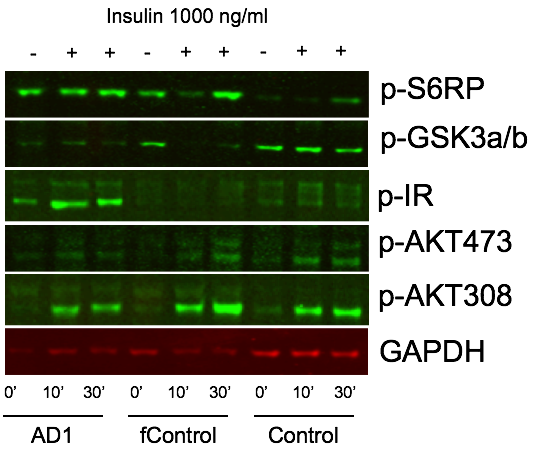


**Figure S1.** Western blots of iPSC-derived basal cholinergic neuroprecursors cell lines. Cells were insulin deprived overnight before the addition 1000 ng/ml of insulin (insulin was not added to time 0’). Lysates were collected at 0, 10, or 30-minute exposure. Quantified western blot data was normalized over Gapdh and expressed as fold change of 0 ng/ml dose. An effect on concentration response was detected by 2-way ANOVA if indicated. These data correspond to results of three independent experiments. ** P<0.01; *** P<.001. (H) Representative blots from a single experiment showing three of the lines.

**
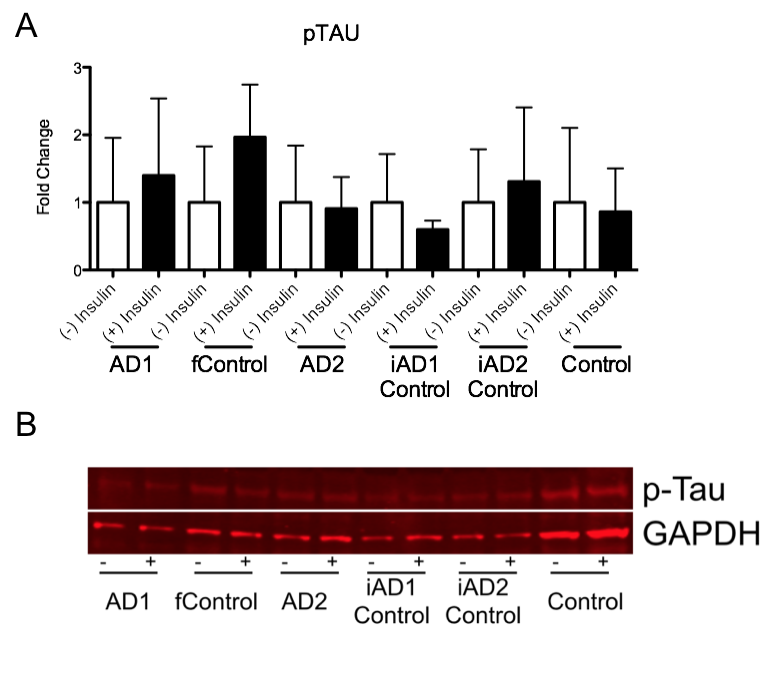
**

**Figure S2.** (A) Western blot quantification of iPSC derived basal cholinergic neurons (two independent experiments). Cells (DIV 34) were insulin deprived for 3 days or given 1000ng/mL every 24 hours for 3 days. (B) Representative blots from a single experiment.
